# Assessment of Patient and Caregiver Attitudes and Approaches to Decision-Making Regarding Bone Marrow Transplant for Sickle Cell Disease: A Qualitative Study

**DOI:** 10.1001/jamanetworkopen.2020.6742

**Published:** 2020-05-29

**Authors:** Nitya Bakshi, Deeksha Katoch, Cynthia B. Sinha, Diana Ross, Maa-Ohui Quarmyne, George Loewenstein, Lakshmanan Krishnamurti

**Affiliations:** 1 Department of Pediatrics, Division of Hematology-Oncology-BMT, Emory University School of Medicine, Atlanta, Georgia; 2 Aflac Cancer and Blood Disorders Center of Children’s Healthcare of Atlanta, Atlanta, Georgia; 3 Department of Social and Decision Sciences, Carnegie Mellon University, Pittsburgh, Pennsylvania

## Abstract

**Importance:**

Bone marrow transplant (BMT) is a potentially curative treatment for sickle cell disease (SCD). Patient and caregiver attitudes toward BMT for SCD and the willingness to accept risks of BMT vary, but these attitudes are not well understood.

**Objective:**

To understand patient and caregiver perceptions of and attitudes toward BMT for SCD and decision-making about BMT.

**Design, Setting, and Participants:**

Qualitative study of interview transcripts from a convenience sample. Transcripts were from adults with SCD and caregivers of patients with SCD recruited from national and regional SCD conferences, symposia, and sickle cell clinics in 2 cities. Interview transcripts were used from the needs assessment phase to develop a patient-decision aid in 2013 to 2014 (group 1) and from the baseline point in 2015 to 2016 (group 2) of the parent trial, a randomized clinical trial of adults and caregivers of patients with SCD to evaluate the effectiveness of a patient decision aid.

**Main Outcomes and Measures:**

Participant perspectives on decision-making regarding BMT for SCD.

**Results:**

Fifty-seven transcripts from adults with SCD and 50 transcripts from caregivers of patients with SCD were included. Median (interquartile range [IQR]) age of adults with SCD was 34 (21-50) years in group 1 and 30 (23-38) years in group 2. The median (IQR) age of caregivers was 42.5 (31-52) years in group 1 and 41 (35-46.5) years in group 2. Most transcripts from adults with SCD (75.0% in group 1 and 72.4% in group 2) and caregivers of patients with SCD (76.7% in group 1 and 85.0% in group 2) were from female participants. Bone marrow transplant was perceived as a treatment option associated with serious risks. Reported attitudes toward BMT occurred on a continuum ranging from unfavorable to favorable. Participants reported serious decisional dilemma regarding BMT for SCD. Most participants expressed interest in learning about BMT or curative treatments.

**Conclusions and Relevance:**

This qualitative study found a continuum in attitudes toward BMT for SCD and highlights the complexity of decision-making in BMT for SCD. Patients and families with SCD expressed an interest in learning about BMT. Future prospective studies of patient decision-making regarding BMT, especially in the context of emerging curative and novel disease-modifying therapies for SCD, are warranted.

## Key Points

**Question:**

What are the attitudes of adult patients with sickle cell disease (SCD) and caregivers of patients with SCD toward bone marrow transplant (BMT) for SCD?

**Findings:**

In this qualitative analysis of 107 interview transcripts of adults with SCD and caregivers of patients with SCD, BMT was viewed as a treatment option associated with significant risks. Wide variation existed in attitudes toward BMT with significant decisional dilemma regarding its use in SCD.

**Meaning:**

Patients with SCD and caregivers of patients with SCD have a range of opinions regarding BMT; future prospective studies of patient decision-making regarding BMT are warranted.

## Teaser

This qualitative study evaluates transcripts of interviews with adults with sickle cell disease and caregivers of patients with sickle cell disease to assess their attitudes and decision-making regarding bone marrow transplantation.

## Introduction

Sickle cell disease (SCD), a chronic multisystem disorder, affects approximately 100 000 people in the US^1^ and is associated with morbidity,^2^ poor health-related quality of life,^3^ and premature mortality.^4,5^ Bone marrow transplant (BMT) is a potential curative therapy.^6^ Factors that may be associated with uptake of BMT include availability of a donor,^7^ perceived risk of morbidity and mortality from BMT,^8^ knowledge of SCD^9^ including long-term complications,^10^ access to BMT clinical trial information,^9^ mistrust of medical professionals,^9^ awareness of BMT as a curative option,^11^ costs associated with BMT, variability in physician philosophy regarding BMT,^12^ and patient and family attitudes toward BMT.^13,14^ While adult patients with SCD,^15,16^ adolescents,^10^ and parents of children with SCD^17^ are willing to accept some risk of BMT-associated morbidity and mortality for the possibility of a cure in a hypothetical scenario, some families with children with sickle cell anemia warranting chronic transfusions decline BMT, even when they have a matched donor.^13^

We sought to understand the perspectives of patients and caregivers toward BMT through qualitative interviews, which have the potential to provide in-depth insights.^18^ This study is a theory-informed secondary analysis of a subset of interview transcripts from a parent study, a study to develop a web-based patient decision aid for therapeutic options in SCD and evaluate its effectiveness in a randomized clinical trial.^19^

## Methods

Study participants included individuals aged 18 years or older with SCD and caregivers of patients with SCD enrolled in the parent study to develop and test a web-based decision aid for SCD.^19^ Participants were drawn from a convenience sample recruited from national and regional conferences, and local clinics in 2 cities. For this analysis, we used interview transcripts from the needs assessment phase in 2013 to 2014 (group 1) and from the baseline of the randomized clinical trial phase in 2015 to 2016 (group 2). Both groups 1 and 2 included adults with SCD and caregivers of pediatric patients, but group 1 also included some caregivers of adult patients. Details related to interview methods and script have been previously published.^19^ We identified all transcripts that contained the terms *bone marrow*, *BMT*, *stem cell*, *transplant*, *bone marrow transplant*, or *cure*. All transcripts that met criteria were included. Those with missing or wrong study ID, missing classification into patient or caregiver groups, interviews in which search terms were out of context or only mentioned by the interviewer, and an interview in which 2 parents participated together were excluded. We also excluded interviews with siblings of adult patients. The final analysis sample consisted of 28 transcripts from adult patients and 30 transcripts from caregivers in group 1, and 29 transcripts from adult patients and 20 transcripts from caregivers in group 2, for a total of 107 transcripts. Interviews used a semistructured format with probes for follow-up, but the exact wording, order, or content of questions may have differed between participants, and some concepts were explored more with some participants than others. The parent study^19^ was approved by the Emory University institutional review board and University of Pittsburgh institutional review board, and participants provided informed consent. This study followed the Standards for Reporting Qualitative Research (SRQR)^20^ reporting guideline for qualitative studies.

We used a content analysis approach to analyze the transcripts which allowed for the conceptual organization of participant narratives. The aim of content analysis was to attain a broad description of the phenomenon; the outcome of the analysis was concepts or categories describing the phenomenon.^21^ Content analysis used both inductive and deductive coding. In inductive coding, categories were derived from the data,^21^ and in deductive coding, categories were developed a priori.^21^ Most categories were developed using deductive coding, based on goals of the study, literature review, and initial review of a small subset of transcripts, and these categories were related to perception of BMT including benefits and risks, attitudes toward BMT, and decision-making regarding BMT. Coding for approximately 70% of all transcripts was performed by one of us (D.K.), then verified by another coder (N.B.) to ensure agreement. The remainder of the transcripts were coded by a single coder (N.B.). After initial coding, an additional category of risk appraisal of BMT was developed by a coder (N.B,), which was related to the category of attitudes toward BMT, and consensus was achieved with 2 other coders (C.S. and L.K.). All analyses were carried out using NVivo software, version 10 (QSR International).

## Results

Fifty-seven transcripts from adults with SCD and 50 transcripts from caregivers of patients with SCD were included. Median (interquartile range [IQR]) age of adults with SCD was 34 (21-50) years in group 1 and 30 (23-38) years in group 2. The median (IQR) age of caregivers was 42.5 (31-52) years in group 1 and 41 (35-46.5) years in group 2. Most transcripts from adults with SCD (75.0% in group 1 and 72.4% in group 2) and caregivers of patients with SCD (76.7% in group 1 and 85.0% in group 2) were from female participants. Demographic data are presented in the Table.

**Table.** Demographic Data

Characteristic	No. (%)			
	Adults with SCD		Caregivers of children or adults with SCD	
	Group 1 (n = 28)	Group 2 (n = 29)	Group 1 (n = 30)	Group 2 (n = 20)
Age, median (IQR), y	34 (21-50)^a^	30 (23-38)	42.5 (31-52)^a^	41 (35-46.5)
Sex				
Male	7 (25)	8 (27.6)	7 (23.3)	3 (15)
Female	21 (75)	21 (72.4)	23 (76.7)	17 (85)
Race				
African American	24 (85.7)	28 (96.5)	25 (83.3)	20 (100)
Other	2 (7.14)	1 (3.5)	2 (6.7)	NA
Missing	2 (7.14)	NA	3 (10)	NA
Ethnicity				
Hispanic	1 (3.6)	3 (10.3)	NA	1 (5)
Non-Hispanic	24 (85.7)	26 (89.7)	25 (83.3)	18 (90)
Missing	3 (10.7)	NA	5 (16.7)	1 (5)
Marital status				
Single	19 (67.9)^b^	26 (89.7)	8 (26.7)	6 (30)
Married	5 (17.9)	2 (6.9)	15 (50)	12 (60)
Divorced/separated/widowed	2 (7.1)	1 (3.4)	4 (13.3)	2 (10)
Missing	2 (7.1)	NA	3 (10)	NA
Educational level				
High school or GED	5 (17.9)	6 (20.7)	9 (30)	NA
Associate degree	NA	2 (6.9)	NA	4 (20)
Some college	11 (39.3)	12 (41.4)	9 (30)	3 (15)
Bachelor degree	4 (14.3)	6 (20.7)	NA	7 (35)
Master degree or doctorate	5 (17.9)	2 (6.9)	7 (23.3)	5 (25)
Other/trade school	1 (3.5)	1 (3.4)	NA	1 (5)
Missing	2 (7.1)	NA	5 (16.7)	NA
Employment				
Full time	6 (21.4)	1 (3.5)	9 (30)	10 (50)
Part time	12 (42.9)	19 (65.5)	2 (6.7)	3 (15)
None	7 (25)	9 (31)	12 (40)	7 (35)
Missing	3 (10.7)	NA	7 (23.3)	NA
Genotype^c^				
HbSS	10 (35.7)	19 (65.5)	16 (53.33)	14 (70)
HbSC	7 (25)	4 (13.8)	4 (13.33)	4 (20)
HbS-β-thalassemia	4 (14.3)	2 (6.9)	1 (3.33)	2 (10)
Other/not known/missing	7 (25)	4 (13.8)	9 (30)	NA
Caregiver type				
Parent	NA	NA	27 (90)	20 (100)
Grandparent	NA	NA	3 (10)	NA

Abbreviations: IQR, interquartile range; NA, not applicable; SCD, sickle cell disease.

^a^ n = 26.

^b^ Includes 3 participants who reported significant other.

^c^ Genotype of child was indicated for caregivers.

### Perception of BMT

When BMT was discussed, participants used terms such “cure,” “get rid of (the disease),” “no longer having sickle cell,” and “free from sickle cell” to describe the advantages of BMT. Some also described the benefits as the ability to “live a normal life,” the “chance to have a normal childhood,” have less or no pain, not “have to worry about taking medicine everyday,” have fewer complications, or not get sick anymore. When discussed, risks associated with BMT were described as being serious, with participants using terms “serious side effects,” “it’s a dangerous operation,” “risky,” and some participants discussed the risk of death or that it “may not work.”

Few mentioned specific complications of BMT such as graft-vs-host disease, infections, or infertility. Some participants expressed inaccurate information, for example, referring to BMT as surgery, or not knowing if BMT would be a cure. Many participants expressed gaps in understanding of BMT, but patient understanding could only be ascertained to the extent discussed in interviews because BMT-related knowledge was not assessed using a structured survey.

### Continuum in the Attitudes Toward BMT

Several participants discussed BMT in neutral terms, or did not offer sufficient information for the authors to ascertain how they appraised risk of BMT. Among those who discussed BMT in sufficient detail, we observed a continuum of attitudes toward BMT, ranging from unfavorable to favorable. Those who described BMT in unfavorable terms tended to focus on the risks of transplant such as death and the possibility of complications of BMT, such as rejection. They used terms such as “never allow it to happen,” being “leery” of BMT, “wouldn’t consider an option for me,” or “I don’t want no bone marrow transplant because it’s that you could die from it.” Individuals who reported that they knew of or had heard of someone who died or had a bad outcome typically described BMT in unfavorable terms. We found that some patients and caregivers perceived a threshold of disease severity beyond which they may consider BMT. Some individuals used terms such as the “last option,” a “last resort,” “(in a) life-or-death” situation, or “if it came to him staying alive we would make that choice.” For others, the threshold was perceived serious worsening of disease status, for example, if they had a “rise in complications,” or “if I was in really bad pain and that was one of my last resources, then maybe….” One parent said about her son, “He’s getting to the point where he’s kind of sick of the pain, and …he wants to do a bone marrow transplant at some point.” For one parent this threshold was failure of other treatments, “Without knowing that it’s going to be 100 percent successful then I would only choose it if I had to, but that’s if the other options failed like hydroxyurea wasn’t working and he was very, very sick and blood transfusions were just starting to take over his whole life.”

At the other end of the spectrum was discussion about BMT in favorable terms. Participants appeared to weigh risks of BMT against the risks associated with continued morbidity and possibility of mortality of SCD. An adult patient said, “I’m all for it if I can there’s some very strong risks to it and it’s not the easiest process but I mean I still believe that it’s a great opportunity for patients with sickle cell.” Another adult patient highlighted the risks of mortality with SCD itself, “Even if it’s (about BMT) life threatening I’d still do it....I would take the chance, because either way it go, living with sickle cell, eventually is life threatening.” And a parent who said, “She can lose her life. But without me doing something, that could happen anyway from sickle cell. So why not try and give her a fighting chance?”

Some participants did not appear to have thought about BMT. One said, “I haven’t looked up any information on that because it’s never crossed my mind, you know.”

We found that self-perception of disease burden appeared to be a factor in the attitude toward BMT for some participants. One participant said, “If my condition isn’t that bad enough to me then I won’t even consider getting it.” Another said, “I don’t think my pain crises are that frequent or bad to have to get a bone marrow transplant.”

Some participants also expressed a fear of exchanging SCD for another unfamiliar disease, indicating that considering BMT and its risks would be like exchanging “something you know, versus something you don’t know.” One participant expressed this concern as “You don’t want to get rid of sickle cell and end up with something worse.” Another talked about how meeting a parent whose child had experienced a complication of transplant had “wished instead of doing the stem cell and the bone marrow transplant she rather for her child to be kept with sickle cell because it’s like you’re trading one end for another one....” One parent also expressed fear with having to think about BMT as a treatment, “But someday I might have to deal with it. and that is my biggest fear.”

### Awareness of End-Organ Complications of SCD

Participants in group 2 were asked an open-ended question regarding the future or long-term complications of SCD that were discussed by their doctors. We reviewed transcripts to identify if end-organ complications had been reported by participants, such as organ damage, kidney complications, chronic pain, pulmonary hypertension, leg ulcers, stroke, retinopathy, avascular necrosis/hip complications, or iron overload.

Among the 17 caregivers who received this question, 11 reported being aware of one or more of the above complications, and stroke was most frequently mentioned. Among adults, 15 of 29 adults reported being aware of one or more long-term complications of SCD, and 4 mentioned being aware of long-term complications but did not specify them. While participants knew of one or more of these long-term complications, they were not aware of the breadth of the possible end-organ complications of SCD.

### Decisional Dilemma Regarding BMT for SCD

We reviewed a subset of 10 transcripts (9 of which were caregivers), in which participants had considered or were considering BMT, had mentioned testing for (human leukocyte antigen) match or were in the process of finding a potential donor. Caregivers discussed challenges in decision-making related to BMT and reported considerable decisional dilemma.

“Well lately with this bone marrow thing, we’ve been talking amongst ourselves on the risks and benefits and looking at my daughter and the state she’s in now, and lookin’ the state she can possibly be in, and so we just try to wait things out and try to make a decision from there. And that’s the tough part. We haven’t come to a solid decision on anything yet.”

“We think about the bone marrow transplant. I’m a bit nervous about it, you know, I don’t want to take a chance that to lose [my child] and we could just, you know, even if he’s uncomfortable, just to take a risk and not him be ok, but I see he’s miserable and he’s uncomfortable and he’s depressed at times and he just does not like feeling like this. And so it pushes us to think, maybe this is something we should consider and if he was able to have the bone marrow transplant earlier, as in more so than later in life, maybe some of the damage to his organs would not be as severe, the sooner you would do it.”

The small possibility of serious or catastrophic complications influenced the consideration of BMT as a potential treatment option for one parent, “So we have always wanted to do it. But at the beginning (it was) the risk of death, even if it was a small percentage. For you, it will only have to be your one child, it doesn’t matter if its 99 percent successful. If your child is the one that dies it doesn’t matter how much other success they had.”

One parent, whose adult child was going to proceed with BMT, highlighted the time taken for decision-making. The parent said, “But [adult child] had to make the ultimate decision, it was presented before her, she had to decide, it was ultimately her decision, because it was presented like 2 years ago, but she said she wasn’t mentally ready to go through that, but now she is.”

### Interest in Learning About BMT

Patients and caregivers reported that they learned about BMT from medical professionals or from other sources, such as the internet, seminars, articles, support groups, or social media. Several adults indicated they did not learn about BMT from their health care professional.

Most participants in this analysis expressed interest to learn more about BMT or curative treatments. Participants mentioned wanting to learn about their eligibility for BMT, the process of donor selection, risks, benefits, and outcomes of BMT, and improvements in quality of life. Some indicated that they wanted to hear about BMT from others who had received BMT.

## Discussion

The goal of this qualitative study was to evaluate attitudes of adults with SCD and caregivers of patients with SCD about their attitudes and decision-making toward BMT. While this study was not designed to determine knowledge of BMT, comments from several participants reflected gaps in knowledge of BMT. Patients and caregivers reported learning about BMT from sources other than their health care professional. This finding is consistent with the observation by Stallings et al^11^ that those who had heard about BMT outnumber those who had discussed it with their physician. A previous study suggests that considerable variation exists among physicians in their philosophies regarding BMT for SCD,^12^ and these individual philosophies may affect whether BMT is discussed or how it is presented. This study presents a rationale for increasing education about all treatment options including BMT because physician discussion of BMT plays a vital role in patients’ and families’ understanding of the curative potential of transplantation in SCD.^11^ As patients and caregivers express an interest in learning more about BMT, education through symposia^22^ and websites^19^ may offer learning experiences about BMT and other treatment options.

Although only a subset of participants was asked about long-term complications of SCD, participants were not aware of the breadth of end-organ complications of SCD. We cannot ascertain if these complications were not discussed by their health care professional or if patients did not recall them. Limited knowledge of SCD complications is consistent with the description by Roth et al,^14^ in which most parents believed their child’s sickle cell would get better, would not prevent them from achieving life goals, and would not shorten their child’s life span. Lack of understanding of the long-term complications of SCD may influence adoption of disease modifying therapies,^12^ and could affect willingness to accept risks of BMT.^10^ Among patients and families of those who underwent BMT for SCD, concerns about the unpredictable onset of complications and worry about the long-term complications of SCD were cited as some of the reasons for BMT.^23^ Hematologists should address potential for end-organ damage and mortality in SCD, and future studies should seek to understand if and how knowledge of potential end-organ complications influences the decision to seek disease-modifying and curative therapies.

In this qualitative analysis, BMT was perceived as a treatment option associated with serious risks. We observed a continuum in the attitudes and risk appraisal of BMT. Previous studies have reported variability in acceptance of risk of morbidity and mortality associated with BMT among adolescents^10^ and adults^15,16^ with SCD, with a number of parents and families not willing to accept any risk of BMT.^10^ Among those who spoke about BMT in favorable terms, determining whether these patients and families would proceed with BMT when making a decision for themselves or their children was difficult because the utility of theoretical risk taking as a surrogate to real-life decision-making is not known,^10^ and decision-making in hypothetical scenarios may differ from decision-making in real-life situations.^24^

The decision to not consider BMT because of its inherent risk may be explained by the preference to avoid risk-seeking behavior when faced with uncertainty, in which outcomes obtained with certainty are over-weighted relative to uncertain outcomes.^25^ Faced with uncertainty, individuals may display risk aversion in choices involving gains, and show preference for “a sure gain rather than a larger gain which is merely probable.”^25^^(p268)^ It is possible that patients with SCD accept their current state of health and display risk aversion toward BMT, a probable larger gain, because the outcome is uncertain. In contrast, in the context of sure losses, people may display “risk-seeking preference for a loss that is merely probable over a smaller loss that is certain.”^25^^(p269)^ It is possible that those with SCD who perceive risks of morbidity and mortality because of SCD consider the possibility of BMT. Treadwell and Lenert^26^ apply the concept of the “reference level” to a health scenario, state that changes in health are not valued according to absolute qualities, and the new health state is valued according to increments (ie, gains) or decrements (ie, losses) relative to the reference level,^26^ which is typically the current state of health.^27^ It is possible that those who perceive their health status as poor value the potential positive value of BMT more than those in self-perceived good health. Winter et al^28^ have described that less healthy people may have a stronger preference for life-prolonging treatments than healthy people. Verma et al^29^ state that patients in poor health may be more likely to accept or seek an aggressive intervention to avoid death even though the intervention may result in discomfort or disability, whereas the patient in good health may not see the value of those interventions. Further research is warranted on the role of these phenomena in the decision-making process for curative therapies in SCD.

Results of this study suggest that patient’s perception of their disease severity appeared to influence attitudes toward BMT. Ubel et al^30^ state that people with chronic illness often adapt physically and emotionally to their health states. It is possible that over time patients may appraise their state of health more favorably than what is ascertained from their descriptions of SCD-related complications. There may also be a “response shift” as proposed by Spranger and Schwartz,^31^ which involves changing of internal standards, values, and conceptualizing of quality of life, and judgments of health may stay stable despite large changes in objective measures of health.^32^ Future studies are warranted to investigate factors associated with risk appraisal and decision-making about BMT.

This study also highlights the complex nature of decision-making regarding BMT in SCD, and when to pursue BMT among patients and caregivers that appear inclined to do so. As BMT is one of the available treatment options and may be associated with morbidity or mortality, treatment discussions should review all available options and account for patient values and preferences, in keeping with the premise of shared decision-making.^33^

### Strengths and Limitations

The strengths of this study include the large sample of adults with SCD as well as caregivers of patients with SCD who were recruited from national and regional meetings and local clinics. The use of qualitative research methods to describe heterogeneity in patient attitudes and decision-making regarding BMT is another strength. This study presents insight into patient and caregiver attitudes that has the potential to guide the efforts to design educational interventions to increase awareness and understanding of potential curative treatments in SCD.

Limitations of this study include the secondary nature of these analyses. Participants were from a convenience sample recruited from national and regional conferences and local clinics, and thus the results of this study may not have been representative of all patients with SCD. The data gathered in both phases of the study were not specifically designed to ascertain attitudes related to BMT, how risk is appraised, and how decisions are made regarding BMT. Therefore, not all aspects of this complex decision-making process could be studied. We did not formally assess knowledge of the important aspects of BMT or their source using a quantitative method, which may have identified specific gaps in patient knowledge of BMT. In this analysis, most patients expressed an interest in learning about BMT; however this finding may reflect the transcript selection strategy and may not be representative of the SCD population. These interviews were conducted between 2013 and 2016, before the publication and dissemination of studies reporting outcomes of BMT from sibling donors in adults with SCD,^34^ from haploidentical donors,^35^ and preliminary studies of gene therapy.^36^ These data also predate studies demonstrating the efficacy of several new drugs for SCD, including L-glutamine,^37^ crizanlizumab^38^ and voxelotor,^39^ which may change how patients view curative therapies, and may add further complexity to the decision-making process.

## Conclusions

Results of this qualitative study suggest a continuum in attitudes toward BMT for SCD, and highlights the complexity of decision-making in BMT for SCD. Patients and families with SCD expressed an interest in learning about BMT. Future prospective studies are warranted regarding patient decision-making about BMT, especially in the context of emerging curative and novel disease-modifying therapies for SCD.
